# Evaluation of the Need for Intensive Care in Children With Pneumonia: Machine Learning Approach

**DOI:** 10.2196/28934

**Published:** 2022-01-27

**Authors:** Yun-Chung Liu, Hao-Yuan Cheng, Tu-Hsuan Chang, Te-Wei Ho, Ting-Chi Liu, Ting-Yu Yen, Chia-Ching Chou, Luan-Yin Chang, Feipei Lai

**Affiliations:** 1 Department of Pediatrics National Taiwan University Hospital, College of Medicine National Taiwan University Taipei City Taiwan; 2 Graduate Institute of Biomedical Electronics and Bioinformatics National Taiwan University Taipei City Taiwan; 3 Taiwan Centers for Disease Control Taipei City Taiwan; 4 Department of Pediatrics Chi Mei Medical Center Tainan City Taiwan; 5 Department of Surgery College of Medicine National Taiwan University Taipei City Taiwan; 6 Institute of Applied Mechanics National Taiwan University Taipei City Taiwan; 7 Department of Civil Engineering National Taiwan University Taipei City Taiwan; 8 Department of Computer Science and Information Engineering National Taiwan University Taipei City Taiwan; 9 Department of Electrical Engineering National Taiwan University Taipei City Taiwan

**Keywords:** child pneumonia, intensive care, machine learning, decision making, clinical index

## Abstract

**Background:**

Timely decision-making regarding intensive care unit (ICU) admission for children with pneumonia is crucial for a better prognosis. Despite attempts to establish a guideline or triage system for evaluating ICU care needs, no clinically applicable paradigm is available.

**Objective:**

The aim of this study was to develop machine learning (ML) algorithms to predict ICU care needs for pediatric pneumonia patients within 24 hours of admission, evaluate their performance, and identify clinical indices for making decisions for pediatric pneumonia patients.

**Methods:**

Pneumonia patients admitted to National Taiwan University Hospital from January 2010 to December 2019 aged under 18 years were enrolled. Their underlying diseases, clinical manifestations, and laboratory data at admission were collected. The outcome of interest was ICU transfer within 24 hours of hospitalization. We compared clinically relevant features between early ICU transfer patients and patients without ICU care. ML algorithms were developed to predict ICU admission. The performance of the algorithms was evaluated using sensitivity, specificity, area under the receiver operating characteristic curve (AUC), and average precision. The relative feature importance of the best-performing algorithm was compared with physician-rated feature importance for explainability.

**Results:**

A total of 8464 pediatric hospitalizations due to pneumonia were recorded, and 1166 (1166/8464, 13.8%) hospitalized patients were transferred to the ICU within 24 hours. Early ICU transfer patients were younger (*P*<.001), had higher rates of underlying diseases (eg, cardiovascular, neuropsychological, and congenital anomaly/genetic disorders; *P*<.001), had abnormal laboratory data, had higher pulse rates (*P*<.001), had higher breath rates (*P*<.001), had lower oxygen saturation (*P*<.001), and had lower peak body temperature (*P*<.001) at admission than patients without ICU transfer. The random forest (RF) algorithm achieved the best performance (sensitivity 0.94, 95% CI 0.92-0.95; specificity 0.94, 95% CI 0.92-0.95; AUC 0.99, 95% CI 0.98-0.99; and average precision 0.93, 95% CI 0.90-0.96). The lowest systolic blood pressure and presence of cardiovascular and neuropsychological diseases ranked in the top 10 in both RF relative feature importance and clinician judgment.

**Conclusions:**

The ML approach could provide a clinically applicable triage algorithm and identify important clinical indices, such as age, underlying diseases, abnormal vital signs, and laboratory data for evaluating the need for intensive care in children with pneumonia.

## Introduction

Despite recent advances in vaccine development, pneumonia remains a major cause of hospitalization and mortality in children in Taiwan and worldwide [1,2]. New pathogens, such as the recent coronavirus causing COVID-19, continue to cause outbreaks of pneumonia and other severe respiratory infections [3,4]. For hospitalized patients with critical conditions, the timely decision to admit them to the intensive care unit (ICU) is crucial for better prognosis and overall medical care quality [5]. The decision is usually made by doctors based on clinical criteria (eg, chief complaint, symptoms/signs, vital signs) and laboratory criteria (eg, microbiology tests, complete blood count, biochemical examinations). However, no well-structured nor quantitative approach exists.

The community-acquired pneumonia management guidelines from the Pediatric Infectious Diseases Society and the Infectious Diseases Society of America [6] recommend that pediatric patients who need ventilation, have low blood pressure, or have low oxygen saturation be admitted to the ICU for pneumonia. Other risk factors, including white blood cell count and hemoglobin, have been associated with exacerbation among pneumonia patients during hospitalization [7]. Some studies have tried to develop clinical scoring systems to standardize prognosis and disease exacerbation evaluations. For example, a modified version of the Sequential Organ Failure Assessment score for children used vital signs (blood pressure, oxygen saturation), laboratory data (creatinine, platelet count), and medications to evaluate the risk of in-hospital mortality [8]. Other scoring systems, such as the Pediatric Early Warning Score (PEWS) and Pediatric Advanced Warning Score, have been proposed to assist the evaluation of deterioration of pediatric inpatients [9-11]. Gold et al [12] used a modified version of PEWS calculated at admission to predict ICU admission and reported an area under the receiver operating characteristic curve (AUC) of 0.86. Nevertheless, the varying sensitivity, specificity, and degrees of human effort limited their clinical application.

In the era of health data science, using large amounts of patient data to develop algorithms to solve clinical problems has become an important approach [13-18]. For example, Makino et al [19] applied a logistic regression model to predict aggravation of diabetic kidney disease 180 days after discharge using patient demographic data, lab tests, diagnosis codes, and medical history. Their model reached an AUC of 0.74 [19]. Studies conducted in the emergency service setting showed promising results in triaging patients with asthma and chronic obstructive pulmonary disease [20]. In the critical care setting, Zhang et al [16] developed an ensemble model for the prediction of agitation in invasive mechanical ventilation patients under light sedation; an automated electronic health records model to identify patients at high risk of acute respiratory failure or death was validated retrospectively and prospectively and was determined to be feasible for real-time risk identification [17]. Artificial intelligence technology is assisting us with interpreting complex data from critical patients such as patients with acute respiratory distress syndrome (ARDS) and enables us to further improve the management of critically ill patients with individual treatment plans [18]. In these studies, machine learning (ML) algorithms were usually implemented because of the strength of incorporating large data sets and exploring the hidden relationships among features [13,14]. The most common type of clinical task (eg, determining whether the patient has a specific diagnosis, the clinical severity, and the prognosis, such as survival after a specific period) was classification. Decision tree–based models usually yield the most promising results in these clinical scenarios because of their strength in classification tasks [14,20,21].

A computer-aided prognosis prediction framework has also been applied to evaluate deterioration of pediatric inpatients. Zhai et al [22] used electronic health records in a single medical center to predict the need for pediatric intensive care within the first 24 hours of admission. Their logistic regression model reached an AUC of 0.91. Mayampurath et al [23] used 6 common vital signs (eg, temperature, pulse, blood pressure) to predict an ICU transfer event up to 36 hours in advance, reaching AUCs of 0.7-0.8. Rubin et al [24] applied a boosted trees model to electronic health records to predict pediatric ICU transfer at most 2 hours to 8 hours in advance with an AUC of 0.85. These deterioration evaluation models showed promising results with general pediatric patients.

Most ML studies for pneumonia patients have focused on using clinical imaging data for diagnosis or mortality [25-27]. Few studies have explored the possibility of developing an ML-based prediction framework to evaluate the need for intensive care among pediatric pneumonia patients and to yield clinically applicable performance. Therefore, we aimed to use clinical data from children with pneumonia to develop ML algorithms to predict the need for ICU transfer within 24 hours of admission, which could support physician decision-making.

## Methods

### Data Source

We enrolled pneumonia patients aged under 18 years admitted to the National Taiwan University Hospital from January 2010 to December 2019. The clinical data for enrolled patients were retrieved from the National Taiwan University Hospital-integrated Medical Database, and all data were de-identified before being analyzed. The institutional review board of the National Taiwan University Hospital approved this study and the use of de-identified electronic health records (201912131RINB).

The diagnosis of pneumonia was determined from the hospital records if both of the following criteria were met: (1) clinical manifestation of respiratory tract infection at admission, including symptoms (eg, dyspnea, rhinorrhea, cough, sputum), abnormal breath sounds (eg, rales, crackles, rhonchi), or a preliminary diagnosis recorded within 24 hours of admission (see Table S1 in Multimedia Appendix 1), and (2) the International Classification of Disease, ninth revision (ICD-9) and tenth revision (ICD-10) diagnostic codes related to pneumonia at discharge (see Table S2 in Multimedia Appendix 1).

### Collection of Clinically Relevant Features

Data including demographics, underlying diseases, vital signs, pathogens, and laboratory data, which were available within 24 hours of hospitalization and prior to ICU transfer, were collected and included in the statistical analysis, model training, and performance evaluation, as seen in Table S3 in Multimedia Appendix 1. Underlying diseases were identified using ICD-9 and ICD-10 codes. The aforementioned clinically relevant features associated with pneumonia prognosis were also selected and ranked by 3 pediatricians specializing in pediatric infectious diseases, with 5, 10, and over 20 years of experience. If missing rates of cohort data were greater than 30%, features were excluded.

### Outcome of Interest

The outcome of interest was ICU admission within 24 hours of hospitalization, including those directly admitted to the ICU from emergency departments or death within 24 hours of hospitalization. Therefore, patients transferred to the ICU after 24 hours of admission were excluded. Readmissions due to pneumonia within 14 days or due to other conditions within 3 days were also excluded because they might be related to previous admission. The cohort was thus categorized into 2 groups: early ICU transfer (ie, patients transferred to the ICU or who died within 24 hours of admission) and no ICU admission (patients who were not admitted to the ICU through discharge).

### Statistical Analysis

In addition to descriptive analyses, we used chi-square tests for categorical variables to compare differences between the early ICU transfer group and the no ICU admission group. For numerical variables, the Shapiro-Wilk test was used to test normality, the Mann-Whitney *U* test was used for between-group comparisons if the data were not normally distributed, and the *t* test was used if data were normally distributed. The Benjamini-Hochberg procedure was applied to adjust for multiple comparisons. Adjusted *P* values <.05 were considered significant.

### Model Training and Performance Evaluation

Based on previous research, we developed a logistic regression model as a baseline reference. Then, we trained random forest (RF) and eXtreme Gradient Boosting (XGB) models because of their promising performance on clinical classification tasks [14,16,17,20,28-31]. For model training, the data set was separated into development and validation sets at a 4:1 ratio via random selection. The ML models were trained using the development set with 5-fold cross-validation. The performance was then evaluated using the independent validation set. The accuracy, sensitivity (recall), specificity, positive predictive value (precision), negative predictive value, AUC, and average precision were calculated to compare different algorithms and thresholds.

We chose 3 points to operationalize the best performing model: the points with the highest Youden index [32], high specificity (0.99), and high sensitivity (0.99), which could be applied in different clinical scenarios. The CI was estimated using bootstrapping methods with 1000 samples.

### Comparison of Feature Importance Between the ML Model and Physicians

With the best-performing model selected using the aforementioned performance evaluation, we further generated the relative feature importance list using Tree Explainer based on Shapley Additive Explanations (SHAP) values [21]. The relative feature importance was also ranked by 3 physicians using a 5-point scale, and the list was generated by sorting clinical features according to the average of importance scores assessed by the physicians. Then, the relative feature importance list from the ML model was compared with the relative importance ranked by the physicians.

### Software

All data were managed using the NumPy (version 1.16.5) and Pandas (version 0.25.1) libraries of the Python programming language version 3.7.4 (Python Software Foundation, Fredericksburg, VA). Statistical analyses were conducted using the SciPy package version 1.3.1 [33]. To train the algorithm, we used Scikit-learn (The Scikit-learn Contributors, version 0.21.3) [34] for logistic regressions and the RF model. The XGBoost package (Version 0.90) was used for the XGB algorithm [35]. The performance evaluation was conducted using the Scikit-learn package. The Tree Explainer was built based on SHAP values [21].

## Results

### Cohort Description and Between-Group Comparison

**Figure 1:**
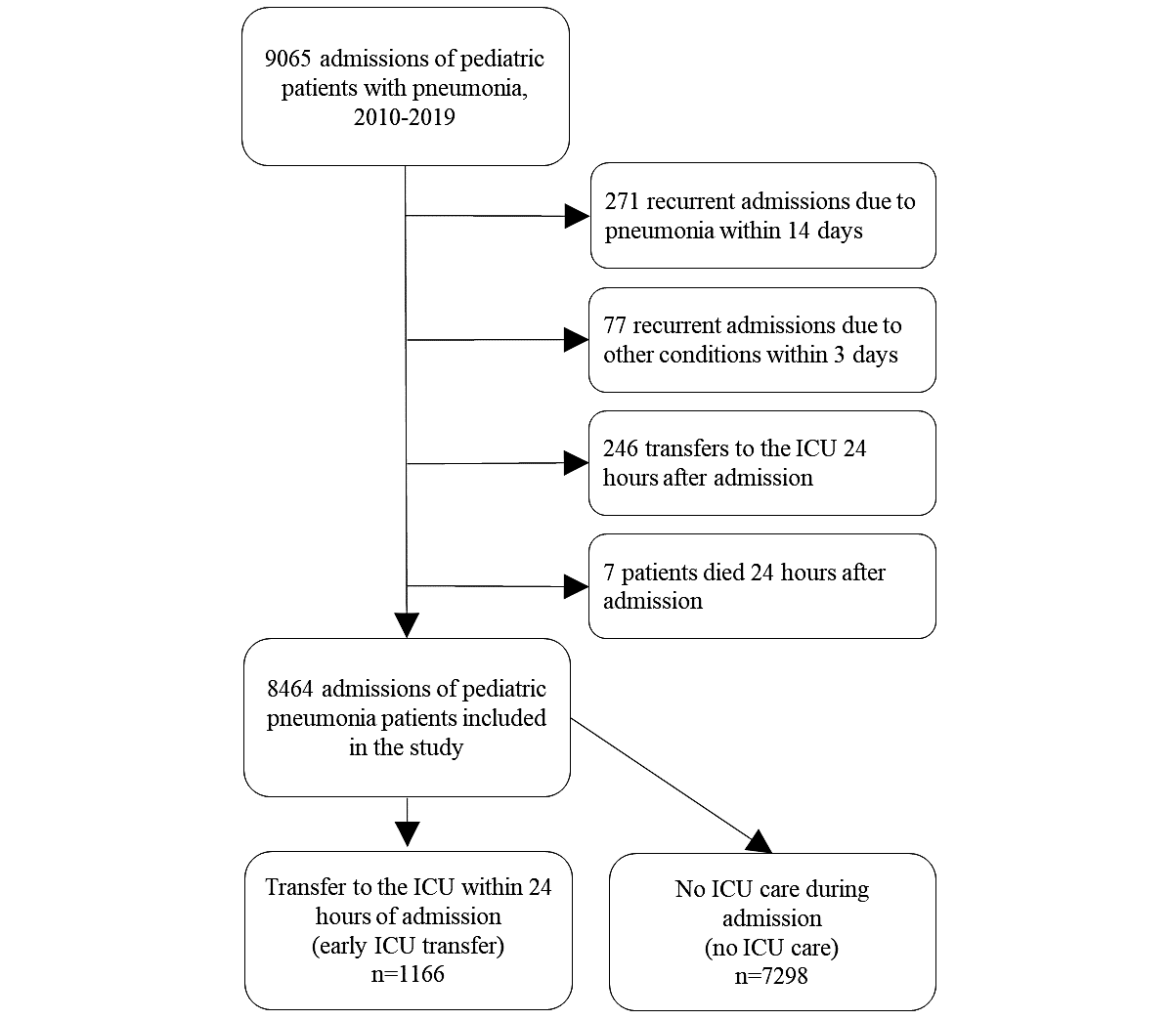
Flowchart of patient enrollment. ICU: intensive care unit.

A total of 6947 patients from 9065 hospitalizations due to pneumonia were included in the study based on their discharge diagnosis code and status at admission. The text mining algorithm correctly labeled 99.8% of admissions with clinical manifestations of a tentative diagnosis using admission notes as examined by the authors using 1000 randomly sampled admissions. Since 601 admissions were excluded based on the aforementioned exclusion criteria, it resulted in a final cohort of 8464 admissions (Figure 1).

The male-to-female ratio was 1.16:1. The median age was 3.1 (IQR 1.7-5.1) years. Among the 8464 admissions included, 1166 admissions (13.8%) were transferred to the ICU or died in the hospital within 24 hours of admission, and they were classified as the early ICU transfer group. The most common underlying disease in the early ICU transfer group was cardiovascular disease (459/1166, 39.4%), followed by neuropsychological disease (416/1166, 35.7%) and congenital anomaly/genetic disorder (310/1166, 26.6%). Common reasons for ICU admission included respiratory failure (566/1166, 48.5%, among which 19.3% [109/566] met the criteria of ARDS), sepsis (392/1166, 33.6%), and chest tube insertion (102/1166, 8.7%). There were 1003 (1003/8464, 11.9%) admissions with a positive microbiological test (as listed in Table S3 in Multimedia Appendix 1) result within 24 hours of admission and prior to ICU transfer. The most commonly identified pathogen at admission was influenza virus type A (14/1166 admissions, 1.2%), followed by influenza virus type B (9/1166 admissions, 0.8%) and *Streptococcus pneumoniae* (5/1166 admissions, 0.4%). Younger age, higher rate of underlying diseases, higher pulse rate, higher breath rate, lower oxygen saturation, lower peak body temperature, and abnormal laboratory data were significantly associated with early ICU transfer (Table 1 and a complete list in Table S4 in Multimedia Appendix 1). However, patients with positive results for influenza A, influenza B, and *S. pneumoniae* at admission were less likely to be transferred to the ICU within 24 hours (*P*=.02, *P*<.001, and *P*<.001, respectively).

**Table 1:** Selective results of clinical feature indices based on early intensive care unit (ICU) transfer.

Features		Early ICU transfer (n= 1166)	No ICU admission (n=7298)	*P* value^a^
**Demographic characteristics**				
	Male, n (%)	623 (53.4)	3916 (53.7)	.89
	Age (years), median (IQR)	2.1 (0.5-5.3)	3.2 (1.8-5.0)	<.001
**Underlying disease^b^**				
	Cardiovascular diseases, n (%)	459 (39.4)	599 (8.2)	<.001
	Neuropsychological diseases, n (%)	416 (35.7)	836 (11.5)	<.001
	CA/GD^c^, n (%)	310 (26.6)	537 (7.4)	<.001
	Respiratory disease, n (%)	228 (19.6)	279 (3.8)	<.001
	Genital-urinary tract disease, n (%)	144 (12.3)	240 (3.3)	<.001
**Vital signs^b^**				
	Lowest pulse (bpm), median (IQR)	136.0 (116.0-152.0)	104.0 (92.0-114.0)	<.001
	Peak body temperature (°C), median (IQR)	37.6 (37.0-38.5)	38.4 (37.6-39.1)	<.001
	Lowest DBP^d^ (mm Hg), median (IQR)	60.0 (51.0-71.0)	66.0 (57.0-75.0)	<.001
	Lowest SBP^e^ (mm Hg), median (IQR)	102.0 (91.0-116.0)	107.0 (97.0-119.0)	<.001
	Initial SBP (mm Hg), median (IQR)	110.0 (98.0-123.0)	112.0 (101.0-124.0)	.001
**Pathogen**				
	Influenza virus type A, n (%)	14 (1.2)	169 (2.3)	.02
	Influenza virus type B, n (%)	9 (0.8)	172 (2.4)	<.001
	*Streptococcus pneumoniae*, n (%)	5 (0.4)	432 (5.9)	<.001
**Lab data^b^**				
	Lymphocyte (%), median (IQR)	21.3 (12.6-36.5)	28.3 (17.2-42.9)	<.001
	Creatinine (U/L), median (IQR)	0.5 (0.3-0.6)	0.4 (0.3-0.5)	<.001
	Segment (%), median (IQR)	67.0 (49.0-79.3)	60.0 (44.4-73.0)	<.001
	CRP^f^ (mg/dL), median (IQR)	1.7 (0.5-5.6)	1.8 (0.6-4.4)	.43
	Hemoglobin (g/dL), median (IQR)	12.7 (11.2-14.0)	12.5 (11.7-13.3)	.02

^a^Adjusted using the Benjamini-Hochberg procedure.

^b^Only the top 5 important features ranked by the Shapley Additive Explanations (SHAP) value are shown. The full table is shown in Table S4 in Multimedia Appendix 1.

^c^CA/GD: congenital anomaly/genetic disorder.

^d^DBP: diastolic blood pressure.

^e^SBP: systolic blood pressure.

^f^CRP: C-reactive protein.

### Model Performance

**Figure 2:**
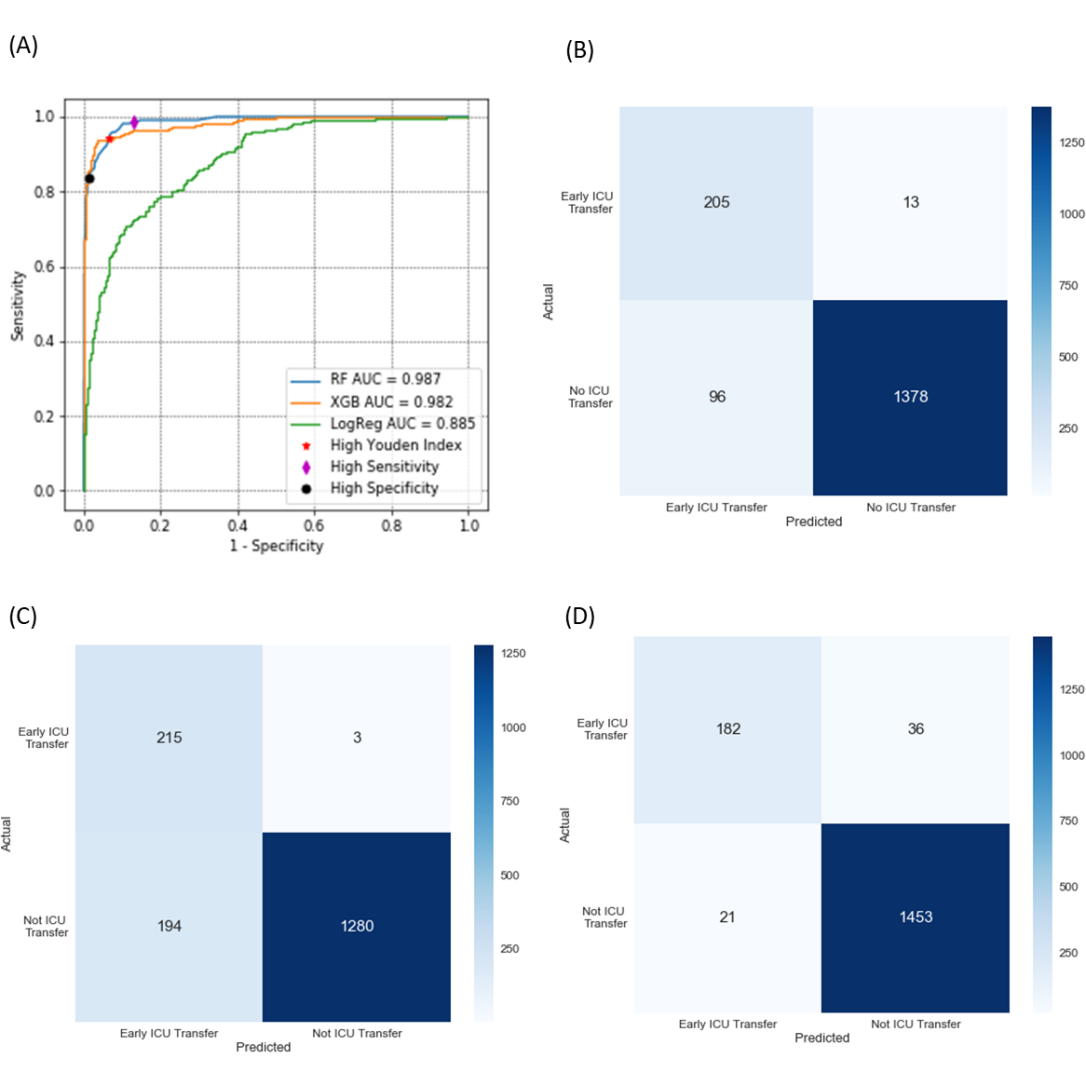
For the early intensive care unit (ICU) transfer and no ICU transfer groups, (A) receiver operating characteristic (ROC) curves and confusion matrices at the operational points with (B) the highest Youden index, (C) 0.99 sensitivity and the highest precision, and (D) 0.99 specificity and the highest sensitivity. AUC: area under the ROC curve; LogReg: logistic regression; RF: random forest; XGB: extreme gradient boosting.

After random selection, 6772 (6772/8464, 80.0%) records were included in the development set, and 1692 (1692/8464, 20.0%) were included in the validation set (Table 2). In the validation set, the RF model achieved the best performance in identifying patients transferred to the ICU within 24 hours after admission (AUC 0.987, 95% CI 0.981-0.992) compared with the XGB model (AUC 0.982, 95% CI 0.972-0.990) and logistic regression model (AUC 0.885, 95% CI 0.863-0.908). The average precision values were 0.932 (95% CI 0.904-0.956) for RF, 0.941 (95% CI 0.917-0.963) for the XGB algorithm, and 0.609 (95% CI 0.543-0.681) for the logistic regression model (Figure 2).

For the RF algorithm, at the point with the highest Youden index, the overall accuracy of the RF algorithm was 0.936 (95% CI 0.930–0.947), sensitivity was 0.940 (95% CI 0.919–0.954), and specificity was 0.935 (95% CI 0.924–0.952; Figure 2). At this threshold, there is approximately one false positive for every 3.1 positive predictions. At the point of highest sensitivity, which could include most patients with early ICU admission with some false alarms, the specificity was 0.868 (95% CI 0.642–0.917), and the negative predictive value was 0.998 (95% CI 0.995-1.000). At the point of highest specificity, which could avoid the most unnecessary ICU admissions, the sensitivity and positive predictive value (precision) for our RF algorithm were 0.835 (95% CI 0.779-0.886) and 0.897 (95% CI 0.883–0.933), respectively.

**Table 2:** Basic characteristics of the development set and validation set.

Characteristics	Development set (n=6772)	Validation set (n=1692)
ICU^a^ transfers or deaths within 24 hours after admission, n (%)	948 (14.0)	218 (12.9)
Unique individuals, n	5581	1576
Length of stay (days), median (IQR)	4.0 (3.0-7.0)	4.0 (3.0-7.0)
Age (years), mean (SD)	4.0 (3.5)	3.9 (3.3)
Male, n (%)	3625 (53.5)	914 (54.0)

^a^ICU: intensive care unit.

### Feature Importance From the ML Algorithm and Clinicians’ Judgment

**Figure 3:**
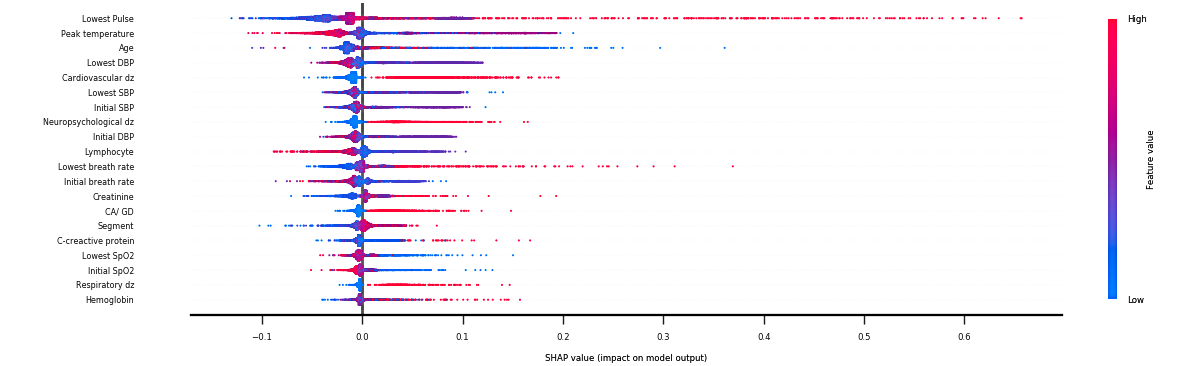
Top 20 important features of the random forest model based on Shapley Additive Explanations (SHAP) values. Every admission data point has one dot on each row for individual features. The color of the dot indicates the value of each feature from the admission data. The pile of dots on the same row to illustrate the density at different SHAP values. CA/GD congenital anomalies/genetic disorder; DBP: diastolic blood pressure; dz: disease; SBP: systolic blood pressure; SpO2: blood oxygen saturation.

Figure 3 shows the top 20 features by relative importance from the RF algorithm based on SHAP values (see Figure S1 in Multimedia Appendix 2 for a complete list). The 5 most important features were lowest pulse rate, peak body temperature, age, lowest diastolic blood pressure, and presence of cardiovascular disease. For physician-rated relative feature importance, the presence of immunodeficiency; lowest oxygen saturation; and presence of solid neoplastic diseases, respiratory diseases, and cardiovascular diseases were considered the most important features (Figure S2 in Multimedia Appendix 3). The presence of cardiovascular diseases, the lowest systolic blood pressure, and the presence of neuropsychological diseases were ranked in the top 10 features with the highest importance measured by both SHAP values in the XGB model and physicians’ judgment.

## Discussion

### Principal Findings

Using the clinical data from 8464 admissions of children with pneumonia, we trained 2 ML algorithms to predict the need for ICU care within 24 hours of admission. Our study showed that ML algorithms could be applied to accurately triage hospitalized pediatric patients with pneumonia and effectively identify those who may need early ICU transfer. The high specificity and sensitivity of our algorithms supported their potential application in real-world clinical scenarios, which could provide a disease-specific alarm for severe conditions with the need for ICU care in a timely manner based on individual patient conditions. Because we only included the available features at admission, this design was considered more practical in clinical use. In addition, the list of feature importance could be explained by the clinical reasoning of human physicians. The explainability further validates the use of the ML approach for the clinical classification task. To our knowledge, our study is the first to explore the possibility of applying ML methods to large clinical data sets for triaging pediatric patients with pneumonia for ICU care.

The identification of a patient with the need for ICU care in the emergency room or in the early stage of the disease might influence medical care quality and clinical outcomes [5,36]. Previous work has revealed the ability to use decision tree–based algorithms to perform classification tasks in clinical scenarios or triage, with some promising preliminary results [13,14,16,17,20,24]. However, applications in clinical classification usually focus on triaging patients with different clinical severities and more general clinical diagnoses, such as respiratory failure, other organ failures, or sepsis [13,14,20,37]. Our work is one of the few studies to focus on a large data set for a specific diagnosis, pediatric pneumonia. Our algorithm’s performance has better performance than previous studies that had AUCs ranging from approximately 0.7 to 0.9 [22-24,29], suggesting the advantage of an ML approach dedicated to children with pneumonia. With satisfactory performance, the application of the ML algorithms we proposed can be applied to support physicians’ decisions for ICU care based on individual patient conditions and further improve health care quality during hospitalization. It can also help reduce clinicians’ burden during outbreaks of community-acquired pneumonia, such as the recent COVID-19 outbreak, or in hospitals with insufficient human resources.

Because we could set up different operational points for the algorithm, our algorithm could be applied in various clinical settings. For example, at the high sensitivity operational point, the specificity could be kept at 0.868 (95% CI 0.642-0.917) with a negative predictive value of 0.998 (95% CI 0.995-1.000), which could be used to rule out those who did not need ICU care. Medical centers accommodating single-digit inpatients with pneumonia per day can operate on this threshold. Using the high sensitivity point, we could help clinicians identify patients who might need ICU admission earlier and reduce the number of undertriaged patients. Although there were one-quarter of the results as false positives, the burden is acceptable when the number of inpatients per day remains low, and false negatives are more harmful. When we further examined the medical records of those false negative cases in the current data set, we found that older age might be related with false negative results. Therefore, clinicians should be aware of false negative results in older children when applying the algorithm for their decision support. In contrast, at the high specificity point (0.99), our algorithm maintained a sensitivity of 0.835 (95% CI 0.779-0.886) and a positive predictive value of 0.897 (95% CI 0.883-0.933). The high specificity with a high positive predictive value suggest that the algorithm could prevent unnecessary ICU admissions, so it may be applied when health care resources are limited or an outbreak happens. Therefore, the algorithm output could be customized according to the clinician’s needs. In this way, the improved discriminability from ML algorithms could contribute to more accurate clinical decision-making and resource allocation. The ML model can not only provide automated estimation in clinical settings but also serve as a tool for training less experienced physicians or setting an alarm in hospitals with fewer human resources. Although the model does not reflect 100% of human physician decisions, it could be considered as a second opinion in the clinical setting and serve as a reference instead of being the only guideline for the final medical decision.

Our study also revealed important clinical feature indices (such as younger age, underlying diseases, higher pulse rate, and lower blood pressure) for the need for early ICU transfer, but patients with positive results for influenza A, influenza B, and *S.* *pneumoniae* at admission were less likely to be transferred to the ICU within 24 hours. These important clinical red flags could help physicians manage critically ill patients. In addition, early detection of the pathogens causing pneumonia in children makes early optimal treatment possible and improves the patient’s clinical condition.

### Limitations

There are some limitations in our study. First, we did not include imaging data, such as chest X-ray images, in our data set. However, diagnosis using the ICD codes relied on the physicians’ clinical judgments, and clinicians might have already considered other clinical clues. Although most pneumonia patients are diagnosed clinically without specific radiological findings, including imaging data might still improve the judgment of clinical severity and thus influence the risk stratification for ICU care. Second, some clinically relevant parameters, such as blood gas values and procalcitonin measures, were not included in our algorithm training because of the high proportion of missing data. Third, the reasons for ICU admission usually varied (eg, ARDS, sepsis, respiratory failure, or other organ failures). Our algorithm could only evaluate the possible needs for ICU admission instead of the clinical diagnosis. With more data collected, an individual algorithm for a specific diagnosis might be developed in the future. Lastly, the algorithms were trained using a data set from a single medical center. Generalizability might be an issue if we would like to apply the findings to other hospital settings. Clinical validation in real-world settings might be required at the next stage to examine the application of ML algorithms in daily clinical work.

### Comparisons With Prior Work

Compared with prior work that evaluated the need for ICU admission for pediatric patients, our disease-specific model for children with pneumonia demonstrated better performance. Our study incorporated up to 41 features from different domains (eg, demographics, vital signs, microbiological tests, and laboratory examinations) with no human-rated components (eg, behavior rating, respiratory difficulty). The strength of our tree-based ML approach is the ability to simultaneously process high-dimensional data linearly or nonlinearly [21]. With ML algorithms, we could integrate data with varying characteristics and solve complicated clinical questions (ie, predict the need for ICU care for hospitalized children with pneumonia). These characteristics enable the ML algorithm to include more clinical data and explore interactions among individual features, which was almost impossible to conduct with human intelligence or traditional statistical approaches, such as logistic regression. To further validate the algorithm’s explainability, we invited 3 experienced physicians to grade the importance of ICU transfer evaluations from a clinical perspective. The results showed that features that were considered to be of higher importance by ML algorithms, such as the lowest systolic blood pressure and the presence of cardiovascular and neuropsychological diseases, were also considered essential features in the physicians’ clinical judgment. The results helped us explain the findings of ML algorithms without being accused of using a “black box” for clinical decision-making. However, some discrepancies were still found. For example, human doctors tend to consider immunodeficiency and solid tumor diseases to be high-risk factors for early ICU transfer, but the importance of these 2 features in the ML algorithms is very low. This discrepancy between machine and human intelligence might be the consequence of proactive management for immunocompromised patients in clinical settings and thus inversely lowers the probability of early ICU admission. When applying the ML algorithm, we still have to consider this limitation in immunocompromised patients and combine the prediction of ML algorithms with clinical judgment. In this way, we could maximize support from machines without neglecting human intelligence.

### Conclusions

In summary, we developed ML algorithms that could accurately classify the risk of early ICU transfer within 24 hours of admission for children with pneumonia. The clinical use of these algorithms might detect high-risk patients earlier and improve the quality of health care for pediatric pneumonia.

## Appendix

Multimedia Appendix 1 Supplementary tables.

Multimedia Appendix 2 Relative feature importance of the random forest model based on Shapley values.

Multimedia Appendix 3 List of feature importance ranked by physicians.

## Abbreviations

ARDS: acute respiratory distress syndrome
AUC: area under the receiver operating characteristic curve
ICD: International Classification of Diseases
ICU: intensive care unit
ML: machine learning
PEWS: Pediatric Early Warning Score
RF: random forest
SHAP: Shapley Additive Explanations
XGB: extreme-gradient boosting
