# Supplementary material for: Evaluation of the Need for Intensive Care in Children With Pneumonia: Machine Learning Approach
Source: JMIR Med Inform. 2022 Jan 27;10(1):e28934. doi: 10.2196/28934 (PMC8832265; doi:10.2196/28934)
Supplement: Multimedia Appendix 1 [file medinform_v10i1e28934_app1.doc]

**Multimedia Appendix 1. Supplementary tables.**

**Table S1***.* Clinical manifestations and tentative diagnosis of respiratory tract infection at admission used as inclusion criteria.

| **Notes** | **Key words** |
| --- | --- |
| Respiratory Tract Infection Related Symptoms | cough, sputum, hoarseness, injected throat, sore throat,  dyspnea/ tachypnea, respiratory distress/ failure,  rhinorrhea, nasal congestion, rhinitis/ coryza  desaturation, cyanosis, chest pain |
| Abnormal Breath Sounds | rales/crackles, wheezes/rhonchi, stridor |
| Tentative diagnosis | pneumonia, empyema, bronchopneumonia, pleural effusion, bronchiolitis, bronchitis, tonsillitis, upper respiratory tract infection, croup, sinusitis, otitis media, rhinitis, pharyngitis, nasopharyngitis, influenza, pleuritis, pleurodynia, laryngitis, hemopneumothorax, hemothorax, ICD codes listed in Table S2 |

**Table S2***.* The ICD9 and ICD10 codes used for pneumonia patients.

| **ICD 9** | | **Description** |  |
| --- | --- | --- | --- |
| 003.22 | | Salmonella pneumonia |  |
| 052.1 | | Varicella (hemorrhagic) pneumonitis |  |
| 055.1 | | Postmeasles pneumonia |  |
| 073.0 | | Ornithosis with pneumonia |  |
| 112.4 | | Candidiasis of lung |  |
| 114.0 | | Primary coccidioidomycosis (pulmonary) |  |
| 114.5 | | Pulmonary coccidioidomycosis, unspecified |  |
| 115.05 | | Pneumonia, infection by histoplasma capsulatum |  |
| 115.15 | | Pneumonia, infection by histoplasma duboisii |  |
| 115.95 | | Pneumonia, infection by histoplasmosis, unspecified |  |
| 130.4 | | Pneumonitis due to toxoplasmosis |  |
| 136.3 | | Pneumocystosis |  |
| 480.0 | | Pneumonia due to adenovirus |  |
| 480.1 | | Pneumonia due to respiratory syncytial virus |  |
| 480.2 | | Pneumonia due to parainfluenza virus |  |
| 480.8 | | Pneumonia due to other virus not elsewhere classified |  |
| 480.9 | | Viral pneumonia, unspecified |  |
| 481 | | Pneumococcal pneumonia |  |
| 482.0 | | Pneumonia due to Klebsiella pneumoniae |  |
| 482.1 | | Pneumonia due to Pseudomonas |  |
| 482.2 | | Pneumonia due to Hemophilus influenzae |  |
| 482.30 | | Pneumonia due to Streptococcus, unspecified |  |
| 482.31 | | Pneumonia due to Streptococcus, Group A |  |
| 482.32 | | Pneumonia due to Streptococcus, Group B |  |
| 482.39 | | Pneumonia due to other Streptococcus |  |
| 482.40 | | Pneumonia due to Staphylococcus, unspecified |  |
| 482.41 | | Pneumonia due to Staphylococcus aureus |  |
| 482.49 | | Pneumonia due to other Staphylococcus |  |
| 482.81 | | Pneumonia due to Anaerobes |  |
| 482.82 | | Pneumonia due to Escherichia coli |  |
| 482.83 | | Pneumonia due to other gram-negative bacteria |  |
| 482.84 | | Legionnaires' disease |  |
| 482.89 | | Pneumonia due to other specified bacteria |  |
| 482.9 | | Bacterial Pneumonia, unspecified |  |
| 483.0 | | Pneumonia due to Mycoplasma pneumoniae |  |
| 483.1 | | Pneumonia due to Chlamydia |  |
| 483.8 | | Pneumonia due to other specified organism |  |
| 484.1 | | Pneumonia in cytomegalic inclusion disease |  |
| 484.3 | | Pneumonia in whooping cough |  |
| 484.5 | | Pneumonia in anthrax |  |
| 484.6 | | Pneumonia in aspergillosis |  |
| 484.7 | | Pneumonia in other systemic mycoses |  |
| 484.8 | | Pneumonia in other infectious diseases classified elsewhere |  |
| 485 | | Bronchopneumonia, organism unspecified |  |
| 486 | | Pneumonia, organism unspecified |  |
| 487.0 | | Influenza with pneumonia |  |
| 511.0 | | Pleurisy, without mention of effusion or current tuberculosis |  |
| 511.1 | | Pleurisy, with effusion, with mention of a bacterial cause other than tuberculosis |  |
| 511.8 | | Pleurisy, other specified forms of effusion, except tuberculous |  |
| 511.9 | | Pleurisy, unspecified pleural effusion |  |
| 513.0 | | Abscess of lung |  |
| 513.1 | | Abscess of mediastinum |  |
| **ICD-10** | | **Description** |  |
| A02.22 | | Salmonella pneumonia |  |
| A37.91 | | Whooping cough, unspecified species with pneumonia |  |
| A48.1 | | Legionnaires' disease |  |
| B01.2 | | Varicella pneumonia |  |
| B05.2 | | Measles complicated by pneumonia |  |
| B25.0 | | Cytomegaloviral pneumonitis |  |
| B37.1 | | Pulmonary candidiasis |  |
| B38.0 | | Acute pulmonary coccidioidomycosis |  |
| B38.2 | | Pulmonary coccidioidomycosis, unspecified |  |
| B39.0 | | Acute pulmonary histoplasmosis capsulati |  |
| B39.1 | | Chronic pulmonary histoplasmosis capsulati |  |
| B39.2 | | Pulmonary histoplasmosis capsulati, unspecified |  |
| B39.5 | | Histoplasmosis duboisii |  |
| B39.9 | | Histoplasmosis, unspecified |  |
| B44.0 | | Invasive pulmonary aspergillosis |  |
| B58.3 | | Pulmonary toxoplasmosis |  |
| B59 | | Pneumocystosis |  |
| J09.X1 | | Influenza due to identified novel influenza A virus with pneumonia |  |
| J10.00 | | Influenza due to other identified influenza virus with unspecified type of pneumonia |  |
| J10.01 | | Influenza due to other identified influenza virus with the same other identified influenza virus pneumonia |  |
| J10.08 | | Influenza due to other identified influenza virus with other specified pneumonia |  |
| J11.00 | | Influenza due to unidentified influenza virus with unspecified type of pneumonia |  |
| J11.08 | | Influenza due to unidentified influenza virus with specified pneumonia |  |
| J12.0 | | Adenoviral pneumonia |  |
| J12.1 | | Respiratory syncytial virus pneumonia |  |
| J12.2 | | Parainfluenza virus pneumonia |  |
| J12.3 | | Human metapneumovirus pneumonia |  |
| J12.81 | | Pneumonia due to SARS-associated coronavirus |  |
| J12.89 | | Other viral pneumonia |  |
| J12.9 | | Viral pneumonia, unspecified |  |
| J13 | | Pneumonia due to Streptococcus pneumoniae |  |
| J14 | | Pneumonia due to Hemophilus influenzae |  |
| J15.0 | | Pneumonia due to Klebsiella pneumoniae |  |
| J15.1 | | Pneumonia due to Pseudomonas |  |
| J15.20 | | Pneumonia due to staphylococcus, unspecified |  |
| J15.211 | | Pneumonia due to Methicillin susceptible Staphylococcus aureus |  |
| J15.29 | | Pneumonia due to other staphylococcus |  |
| J15.3 | | Pneumonia due to streptococcus, Group B |  |
| J15.4 | | Pneumonia due to other streptococci |  |
| J15.5 | | Pneumonia due to Escherichia coli |  |
| J15.6 | | Pneumonia due to other aerobic Gram-negative bacteria |  |
| J15.7 | | Pneumonia due to Mycoplasma pneumoniae |  |
| J15.8 | | Pneumonia due to other specified bacteria |  |
| J15.9 | | Unspecified bacterial pneumonia |  |
| J16.0 | | Chlamydial pneumonia |  |
| J16.8 | | Pneumonia due to other specified infectious organisms |  |
| J17 | | Pneumonia in diseases classified elsewhere |  |
| J18.0 | | Bronchopneumonia, unspecified organism |  |
| J18.1 | | Lobar pneumonia, unspecified organism |  |
| J18.8 | | Other pneumonia, unspecified organism |  |
| J18.9 | | Pneumonia, unspecified organism |  |
| J85.0 | | Gangrene and necrosis of lung |  |
| J85.1 | | Abscess of lung with pneumonia |  |
| J85.2 | | Abscess of lung without pneumonia |  |
| J85.3 | | Abscess of mediastinum |  |
| J86.9 | | Pyothorax without fistula |  |
| J90 | | Pleural effusion, not elsewhere classified |  |
| J91.8 | | Pleural effusion in other conditions classified elsewhere |  |
| J92.0 | | Pleural plaque with presence of asbestos |  |
| J92.9 | | Pleural plaque without asbestos |  |
| J94.2 | | Hemothorax |  |
| J94.8 | | Other specified pleural conditions |  |
| J94.9 | Pleural condition, unspecified | | |
| R09.1 | Pleurisy | | |

**Table S3.** Feature selection and preprocessing.

| **Features** | **Notes** | **Preprocessing** |
| --- | --- | --- |
| **Demographic** |  | |
| Sex | Male/ Female | **-** |
| Age | Age at admission in years |  |
| **Underlying Disease** |  | |
| Allergy | With/without the disease, based on diagnosis code at discharge | If there is inconsistency between admissions of the same patient, data were preprocessed such that the same patient is considered having the underlying disease once diagnosed. |
| Autoimmune Disease |  |  |
| Congenital Anomaly/ Genetic Disorder |  |  |
| Cardiovascular Disease |  |  |
| Endocrinologic Disease |  |  |
| Gastrointestinal Disease |  |  |
| Genital-Urinary Tract Disease |  |  |
| Hematological Disease |  |  |
| Hepatobiliary Tract Disease |  |  |
| Immunodeficiency |  |  |
| Malnutrition |  |  |
| Neuropsychologic Disease |  |  |
| Prematurity |  |  |
| Respiratory Disease |  |  |
| Solid Neoplastic Disease |  |  |
| **Vital Sign** |  | |
| Body Temperature | Initial value reported within admission +/- 24 hours and before ICU admission | Readings above the 99th percentile or below the 1st percentile were excluded. Missing data replaced by item-wise median. Minimum, maximum, average, initial values of each item were calculated for feature selection. |
| Pulse |  |  |
| Systolic Pressure |  |  |
| Diastolic Pressure |  |  |
| Breath Rate |  |  |
| Oxygen Saturation |  |  |
| **Pathogen** |  | |
| Influenza Virus Type A | Sampled within admission -7/+1 days, report released within admission -7/+1 days and before ICU admission. | Patients with no positive exam results were considered not having the pathogen. |
| Influenza Virus Type B |  |  |
| *S. pneumoniae* |  |  |
| Parainfluenza |  | Excluded, report released typically after 24H of admission |
| Adenovirus |  |  |
| Respiratory Syncytial Virus |  |  |
| Mycoplasma |  |  |
| *A. baumannii* |  |  |
| *E. coli* |  |  |
| *Gram Negative Bacteria*  *(incl. S. maltophilia*, *P. aeruginosa*, *B. cepacia*) |  |  |
| *H. influenzae* |  |  |
| *M. catarrhalis* |  |  |
| *K. pneumoniae* |  |  |
| *S. aureus* |  |  |
| **Lab Blood Test** |  | |
| White Blood Cell Count | Initial value reported within admission +/- 24 hours and before ICU admission | Missing data replaced by item-wise median. |
| Band Neutrophil |  |  |
| Segmented Neutrophils |  |  |
| Lymphocyte |  |  |
| Hemoglobin |  |  |
| Platelet |  |  |
| C-Reactive Protein (CRP) |  |  |
| Creatinine |  |  |
| Alanine Aminotransferase (ALT) |  |  |
| Sodium |  |  |
| Potassium |  | Readings above the 99th percentile were excluded because it might be cause by hemolysis. Missing data replaced by item-wise median. |

**Table S4*.* Results of Clinical Feature Indices on early ICU transfer.**

| **Features** | Early ICU Transfer  (N = 1,166) | No ICU Admission (N = 7,298) | *P* value^a^ |
| --- | --- | --- | --- |
| **Demographic characteristics** |  |  |  |
| Male (%) | 623 (53.4) | 3,916 (53.7) | .89 |
| Age, median (IQR), y | 2.1 (0.5-5.3) | 3.2 (1.8-5.0) | <.001 |
| **Underlying Disease** |  |  |  |
| Allergy, count (%) | 113 (9.7) | 1,373 (18.8) | <.001 |
| Autoimmune Disease, count (%) | 37 (3.2) | 102 (1.4) | <.001 |
| Congenital Anomaly/Genetic Disorder, count (%) | 310 (26.6) | 537 (7.4) | <.001 |
| Cardiovascular Disease, count (%) | 459 (39.4) | 599 (8.2) | <.001 |
| Endocrinologic Disease, count (%) | 72 (6.2) | 134 (1.8) | <.001 |
| Gastrointestinal Disease, count (%) | 181 (15.5) | 530 (7.3) | <.001 |
| Genital-Urinary Tract Disease, count (%) | 144 (12.3) | 240 (3.3) | <.001 |
| Hematological Disease, count (%) | 139 (11.9) | 343 (4.7) | <.001 |
| Hepatobiliary Tract Disease, count (%) | 46 (3.9) | 79 (1.1) | <.001 |
| Immunodeficiency, count (%) | 77 (6.6) | 243 (3.3) | <.001 |
| Malnutrition, count (%) | 9 (0.8) | 21 (0.3) | .01 |
| Neuropsychological Disease, count (%) | 416 (35.7) | 836 (11.5) | <.001 |
| Prematurity, count (%) | 80 (6.9) | 26 (0.4) | <.001 |
| Respiratory Disease, count (%) | 228 (19.6) | 279 (3.8) | <.001 |
| Solid Neoplastic Disease, count (%) | 30 (2.6) | 90 (1.2) | <.001 |
| **Vital Sign** |  |  |  |
| Peak Body Temperature, °C, median (IQR) | 37.6 (37.0-38.5) | 38.4 (37.6-39.1) | <.001 |
| Higher than normal, count (%) | 199 (40.9) | 4,686 (64.5) | <.001 |
| Normal, count (%) | 271 (55.8) | 2,532 (34.9) |
| Lower than normal, count (%) | 16 (3.3) | 45 (0.6) |
| Lowest Pulse, bpm, median (IQR) | 136.0 (116.0-152.0) | 104.0 (92.0-114.0) | <.001 |
| Higher than normal, count (%) | 322 (61.7) | 670 (9.2) | <.001 |
| Normal, count (%) | 200 (38.3) | 6,518 (89.8) |
| Lower than normal, count (%) | 0 (0.0) | 74 (1.0) |
| Initial Systolic Pressure, mm Hg, median (IQR) | 110.0 (98.0-123.0) | 112.0 (101.0-124.0) | .001 |
| Higher than normal, count (%) | 245 (50.7) | 3,776 (53.7) | .07 |
| Normal, count (%) | 169 (35.0) | 2,491 (35.4) |
| Lower than normal, count (%) | 69 (14.3) | 768 (10.9) |
| Lowest Systolic Pressure, mm Hg, median (IQR) | 102.0 (91.0-116.0) | 107.0 (97.0-119.0) | <.001 |
| Higher than normal, count (%) | 182 (37.7) | 3,079 (43.8) | <.001 |
| Normal, count (%) | 175 (36.2) | 2,721 (38.7) |
| Lower than normal, count (%) | 126 (26.1) | 1,235 (17.6) |
| Initial Diastolic Pressure, mm Hg, median (IQR) | 66.0 (56.0-78.0) | 70.0 (61.0-79.0) | <.001 |
| Higher than normal, count (%) | 169 (34.8) | 2,703 (38.5) | <.001 |
| Normal, count (%) | 197 (40.6) | 3,143 (44.8) |
| Lower than normal, count (%) | 119 (24.5) | 1,175 (16.7) |
| Lowest Diastolic Pressure, mm Hg, median (IQR) | 60.0 (51.0-71.0) | 66.0 (57.0-75.0) | <.001 |
| Higher than normal, count (%) | 109 (22.5) | 2,031 (28.9) | <.001 |
| Normal, count (%) | 196 (40.4) | 3,115 (44.4) |
| Lower than normal, count (%) | 180 (37.1) | 1,875 (26.7) |
| Initial Breath Rate, bpm, median (IQR) | 30.0 (25.0-38.0) | 28.0 (24.0-36.0) | <.001 |
| Higher than normal, count (%) | 308 (58.6) | 4,138 (57.0) | <.001 |
| Normal, count (%) | 189 (35.9) | 3,010 (41.4) |
| Lower than normal, count (%) | 29 (5.5) | 114 (1.6) |
| Lowest Breath Rate, bpm, median (IQR) | 30.0 (24.0-36.0) | 24.0 (20.0-28.0) | <.001 |
| Higher than normal, count (%) | 274 (52.1) | 1,457 (20.1) | <.001 |
| Normal, count (%) | 217 (41.3) | 5,413 (74.5) |
| Lower than normal, count (%) | 35 (6.7) | 392 (5.4) |
| Initial SpO2, %, median (IQR) | 95.0 (91.0-97.0) | 97.0 (95.0-98.0) | <.001 |
| Normal, count (%) | 313 (65.3) | 6,050 (90.9) | <.001 |
| Lower than normal, count (%) | 166 (34.7) | 606 (9.1) |  |
| Lowest SpO2, %, median (IQR) | 94.0 (90.0-97.0) | 96.0 (94.0-97.0) | <.001 |
| Normal, count (%) | 259 (54.1) | 5,397 (81.1) | <.001 |
| Lower than normal, count (%) | 220 (45.9) | 1,259 (18.9) |  |
| **Pathogen** |  |  |  |
| Influenza Virus Type A, count (%) | 14 (1.2) | 169 (2.3) | .02 |
| Influenza Virus Type B, count (%) | 9 (0.8) | 172 (2.4) | <.001 |
| *Streptococcus pneumoniae*, count (%) | 5 (0.4) | 432 (5.9) | <.001 |
| **Lab Data** |  |  |  |
| White Blood Cell Count, k/μL, median (IQR) | 11.8 (8.2-17.0) | 10.2 (7.1-14.2) | <.001 |
| Higher than normal, count (%) | 283 (41.3) | 2,137 (30.1) | <.001 |
| Normal, count (%) | 343 (50.1) | 4,433 (62.5) |
| Lower than normal, count (%) | 59 (8.6) | 519 (7.3) |
| Band Neutrophil, %, median (IQR) | 0.0 (0.0-0.0) | 0.0 (0.0-1.0) | .08 |
| Higher than normal, count (%) | 83 (13.2) | 1,011 (14.3) | .43 |
| Normal, count (%) | 547 (86.8) | 6,042 (85.7) |
| Segment, %, median (IQR) | 67.0 (49.0-79.3) | 60.0 (44.4-73.0) | <.001 |
| Higher than normal, count (%) | 212 (36.7) | 1,460 (22.7) | <.001 |
| Normal, count (%) | 358 (62.0) | 4,830 (75.2) |
| Lower than normal, count (%) | 7 (1.2) | 135 (2.1) |
| Lymphocyte, %, median (IQR) | 21.3 (12.6-36.5) | 28.3 (17.2-42.9) | <.001 |
| Higher than normal, count (%) | 2 (0.3) | 41 (0.6) | <.001 |
| Normal, count (%) | 372 (59.4) | 5,206 (73.8) |
| Lower than normal, count (%) | 252 (40.3) | 1,805 (25.6) |
| Hemoglobin, g/dL, median (IQR) | 12.7 (11.2-14.0) | 12.5 (11.7-13.3) | .02 |
| Higher than normal, count (%) | 310 (39.7) | 2,358 (33.1) | <.001 |
| Normal, count (%) | 382 (48.9) | 4,499 (63. 2) |
| Lower than normal, count (%) | 89 (11.4) | 264 (3.7) |
| Platelet, k/μL, median (IQR) | 281.0 (207.0-370.5) | 261.0 (206.0-330.0) | <.001 |
| Higher than normal, count (%) | 81 (11.9) | 437 (6.2) | <.001 |
| Normal, count (%) | 462 (68.0) | 5,359 (76.4) |
| Lower than normal, count (%) | 136 (20.0) | 1,217 (17.4) |
| CRP, mg/dL, median (IQR) | 1.7 (0.5-5.6) | 1.8 (0.6-4.4) | .43 |
| Higher than normal, count (%) | 315 (59.9) | 4,464 (65.5) | .01 |
| Normal, count (%) | 211 (40.1) | 2,352 (34.5) |
| Creatinine, U/L, median (IQR) | 0.5 (0.3-0.6) | 0.4 (0.3-0.5) | <.001 |
| Higher than normal, count (%) | 22 (3.8) | 15 (0.2) | <.001 |
| Normal, count (%) | 561 (96.2) | 6,771 (99.8) |
| ALT, U/L, median (IQR) | 19.0 (12.5-31.0) | 14.0 (11.0-20.0) | <.001 |
| Higher than normal, count (%) | 76 (14.0) | 409 (6.6) | <.001 |
| Normal, count (%) | 467 (86.0) | 5,825 (93.4) |
| Sodium, mmol/L, median (IQR) | 136.0 (133.4-139.0) | 136.0 (134.0-137.9) | .01 |
| Higher than normal, count (%) | 12 (1.7) | 31 (0.5) | <.001 |
| Normal, count (%) | 391 (54.4) | 3,467 (52.0) |
| Lower than normal, count (%) | 316 (43.9) | 3,173 (47.6) |
| Potassium, mmol/L, median (IQR) | 4.4 (3.9-5.0) | 4.2 (3.8-4.6) | <.001 |
| Higher than normal, count (%) | 136 (19.4) | 628 (9.5) | <.001 |
| Normal, count (%) | 504 (71.9) | 5,423 (82.1) |
| Lower than normal, count (%) | 61 (8.7) | 556 (8.4) |

ICU, intensive care unit; SpO_2_, oxygen saturation measured by pulse oximeter; CRP, C-Reactive Protein; ALT, alanine aminotransferase;

^a^Adjusted with the Benjamini-Hochberg procedure.
