# Supplementary figures and images for: Evaluation of the Need for Intensive Care in Children With Pneumonia: Machine Learning Approach
Source: JMIR Med Inform. 2022 Jan 27;10(1):e28934. doi: 10.2196/28934 (PMC8832265; doi:10.2196/28934)

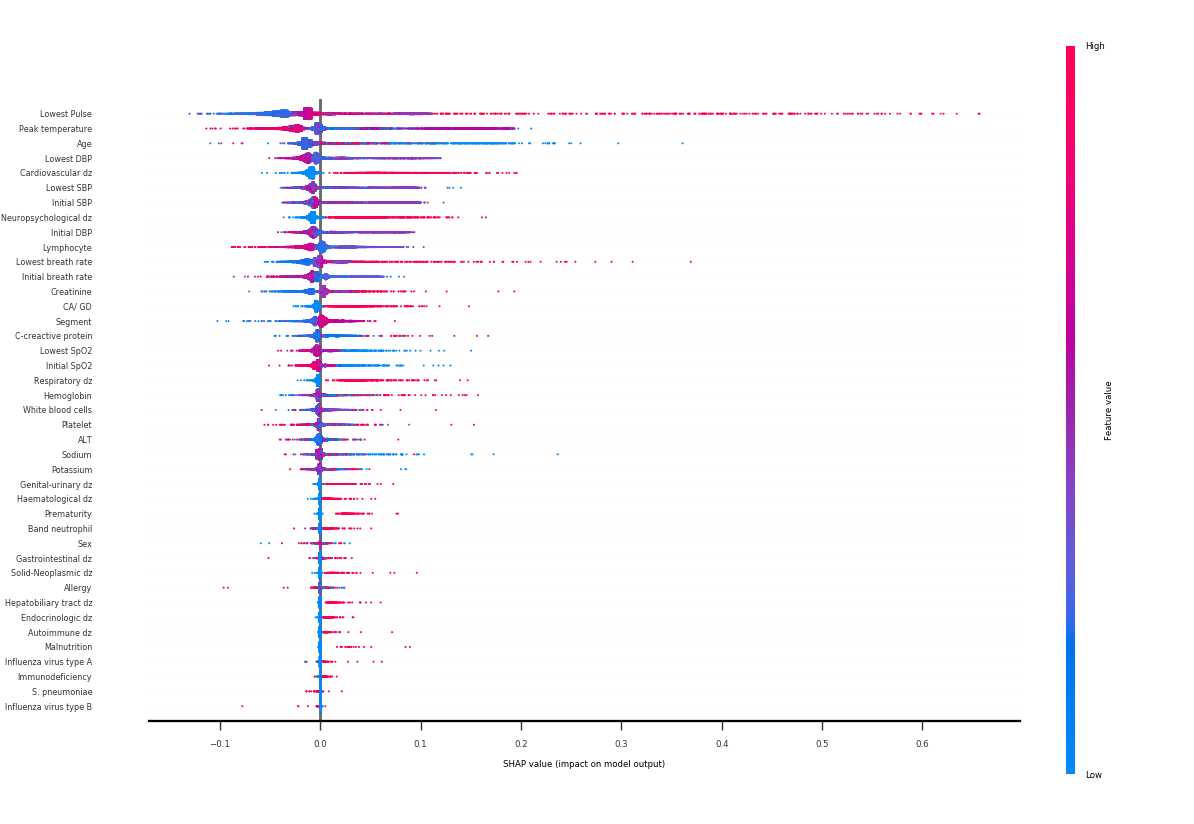

Supplement: Multimedia Appendix 2 [file medinform_v10i1e28934_app2.png]

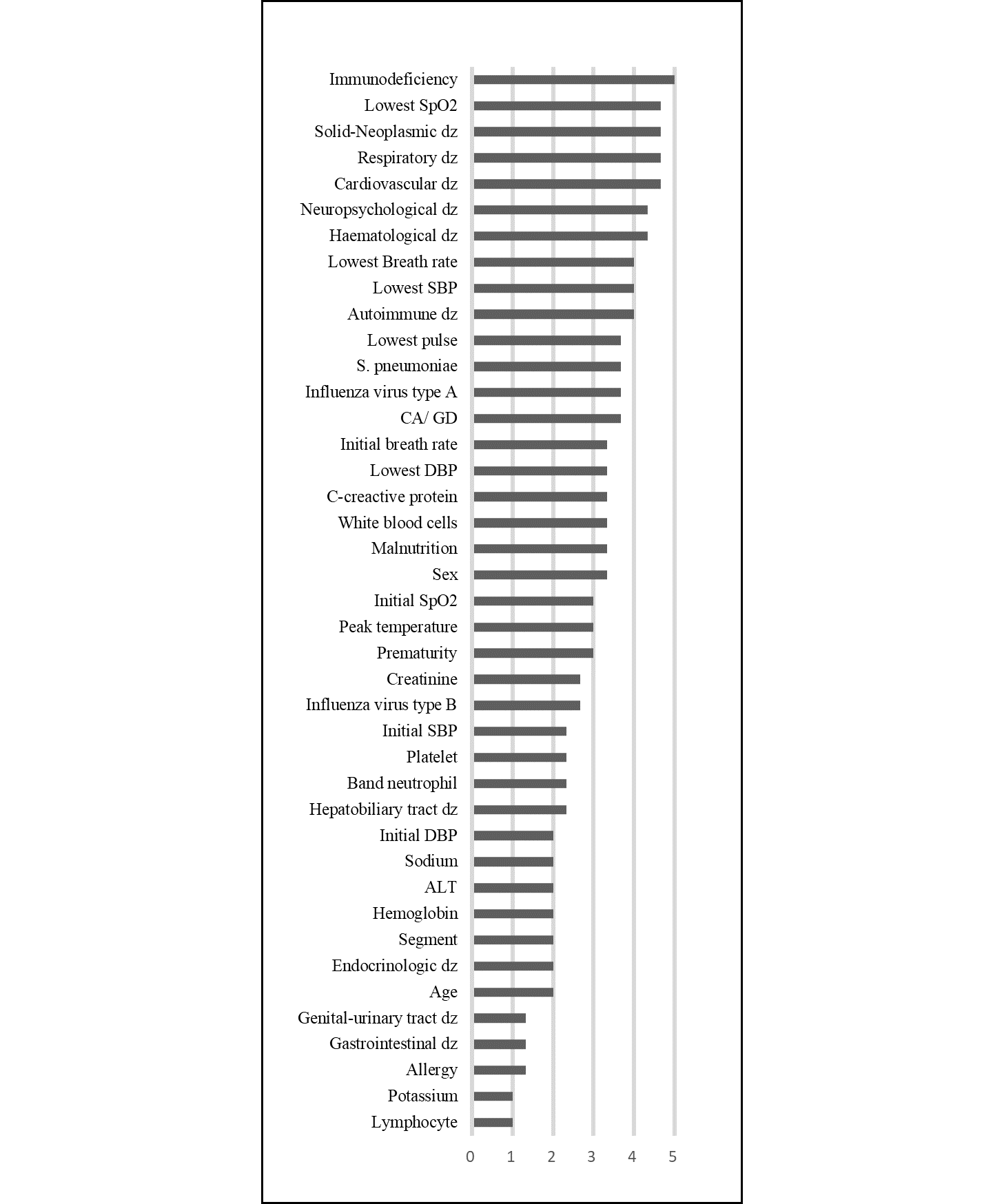

Supplement: Multimedia Appendix 3 [file medinform_v10i1e28934_app3.png]
